# Sensory Neuron-Specific Deletion of Tropomyosin Receptor Kinase A (*TrkA*) in Mice Abolishes Osteoarthritis (OA) Pain via NGF/TrkA Intervention of Peripheral Sensitization

**DOI:** 10.3390/ijms232012076

**Published:** 2022-10-11

**Authors:** InSug O-Sullivan, Ranjan Kc, Gurjit Singh, Vaskar Das, Kaige Ma, Xin Li, Fackson Mwale, Gina Votta-Velis, Benjamin Bruce, Arivarasu Natarajan Anbazhagan, Andre J. van Wijnen, Hee-Jeong Im

**Affiliations:** 1 Biomedical Engineering, University of Illinois at Chicago, Chicago, IL 60612, USA; 2 Ocugen Inc., Malvern, PA 19355, USA; 3 Departments of Anesthesiology, Rush University, Chicago, IL 60612, USA; 4 Surgery, McGill University, Montreal, QC H3T 1E2, Canada; 5 Anesthesiology, University of Illinois at Chicago, Chicago, IL 60612, USA; 6 Jesse Brown Veterans Affairs Medical Center, Chicago, IL 60612, USA; 7 Department of Medicine, University of Illinois at Chicago, Chicago, IL 60612, USA; 8 Biochemistry, University of Vermont, Burlington, VT 05405, USA

**Keywords:** osteoarthritis, NGF/TrkA signaling, pain transportation, hyperalgesia, cellular plasticity

## Abstract

Tropomyosin receptor kinase A (TrkA/NTRK1) is a high-affinity receptor for nerve growth factor (NGF), a potent pain mediator. NGF/TrkA signaling elevates synovial sensory neuronal distributions in the joints and causes osteoarthritis (OA) pain. We investigated the mechanisms of pain transmission as to whether peripheral sensory neurons are linked to the cellular plasticity in the dorsal root ganglia (DRG) and are critical for OA hyperalgesia. Sensory neuron-specific deletion of *TrkA* was achieved by tamoxifen injection in 4-week-old *TrkA^fl/fl^;Na_V_1.8^CreERT2^* (*Ntrk1 ^fl/fl^;Scn10a^CreERT2^*) mice. OA was induced by partial medial meniscectomy (PMM) in 12-week-old mice, and OA-pain-related behavior was analyzed for 12 weeks followed by comprehensive histopathological examinations. OA-associated joint pain was markedly improved without cartilage protection in sensory-neuron-specific conditional *TrkA* knock-out (cKO) mice. Alleviated hyperalgesia was associated with suppression of the NGF/TrkA pathway and reduced angiogenesis in fibroblast-like synovial cells. Elevated pain transmitters in the DRG of OA-induced mice were significantly diminished in sensory-neuron-specific *TrkA* cKO and global *TrkA* cKO mice. Spinal glial activity and brain-derived neurotropic factor (BDNF) were significantly increased in OA-induced mice but were substantially eliminated by sensory-neuron-specific deletion. Our results suggest that augmentation of NGF/TrkA signaling in the joint synovium and the peripheral sensory neurons facilitate pro-nociception and centralized pain sensitization.

## 1. Introduction

Osteoarthritis (OA) is characterized by progressive, degenerative joint damage involving articular cartilage deformation of joint structure and severe pain. OA is a global disease and is a leading cause of disability that affects over 300 million worldwide [1]. Despite the availability of pharmacological and non-pharmacological options, persistent pain associated with OA pain is a debilitating condition that impairs the quality of life and causes heavy burdens on the healthcare system [2,3]. In the United Sates, one out of five individuals suffer chronic pain, and chronic pain affects more people than cardiovascular diseases (CVDs), cancer, and diabetes combined [4,5,6]. Therefore, understanding the mechanisms of pain transmission is the foremost important step for identification of molecular targets that can be leveraged to treat OA, in addition to finding strategies to mitigate or halt progressive joint degeneration.

In the early stages of OA, both meniscal and hyaline articular cartilage are damaged, followed by architectural alterations of the subchondral bone. As OA progresses, synovitis and cartilage erosion can occur, physiological cartilage remodeling and repair follow, and the subchondral bone responds biologically by thickening [7]. As OA advances, nerve growth factor (NGF) and other factors such as brain-derived neurotrophic factor (BDNF); prostaglandins; cytokines including interleukin-1β (IL-1β/IL1B) and tumor necrosis factor-α (TNF-α/TNF); and chemokines, neuropeptides, and calcitonin gene-related peptide (CGRP, CALCA) are released [8]. Pain signaling from the periphery can sensitize further to develop chronic pain [9,10]. Interestingly, hyperalgesia is not strictly correlated with the degree of cartilage degeneration in OA [11].

NGF is not only critical for the growth and survival of neurons, but also plays a significant role in pain transmission and sensation [12]. Preclinical and clinical trials with monoclonal antibodies targeting NGF to treat chronic OA pain, low back pain, and cancer pain have been considered [13]. Studies using a monoclonal antibody against NGF (i.e., tanezumab) reached phase II and III clinical trials but these trials were challenged due to severe adverse effects [14].

NGF binds to NGF receptors, promoting multiple pain signaling pathways, leading to nociceptive pain [15]. TrkA is a cognate receptor for NGF [16] and mutations in the TrkA receptor are associated with congenital insensitivity to pain with anhidrosis (CIPA) or Charcot syndrome [17,18]. *TrkA* mutations can change protein folding, retention in the endoplasmic reticulum (ER), and/or cause aggregation [19]. Different ER quality control systems then degrade TrkA proteins with distinct mutations with different kinetics. These molecular effects combined with disruption of mitochondrial homeostasis and autophagy ultimately result in neurodegeneration. Blocking TrkA with its inhibitor AR786 alleviates OA pain in rats [20,21], and several studies have examined human and experimental animal models to understand the causative mechanisms of OA pain [22].

One line of investigation has explored the concept that alteration of sensory neurons [23] and functional modulation of sensory neurons in symptomatic OA tissues [24] reduces OA pain. Even though the mechanism, efficacy, and safety of OA therapy have been extensively investigated, more effective and definitive therapeutic strategies have not yet emerged.

Here, we investigated the pathological role of the NGF/TrkA pathway in OA-related pain transmission after partial medial meniscectomy (PMM) [25] at 12 weeks of age in three distinct mouse models with a conditional deletion of the NGF receptor TrkA. We comprehensively analyzed and compared with a tamoxifen inducible conditional deletion of *TrkA* in the peripheral sensory neurons (*TrkA^fl/fl^;Na_V_1.8^CreERT2^*), a global deletion (*TrkA^fl/fl^;Rosa^CreERT2^*), and a cartilage-specific deletion (*TrkA^fl/fl^;Aggrecan^CreERT2^*) in mice. Our main results show that sensory-neuron-specific deletion of *TrkA* significantly reduces OA-associated joint pain, spinal glial activity, and central pain transmitters but does not preserve cartilage integrity. The mitigation of hyperalgesia is attributable to the suppression of the NGF/TrkA pathway in fibroblast-like synovial cells and synovial angiogenesis. Our findings support the hypothesis that NGF/TrkA signaling in the joint synovium towards peripheral sensory neurons facilitates pro-nociception and pain centralization.

## 2. Results

### 2.1. Global Conditional Deletion of TrkA in Mice Showed Insensitivity to OA Pain but Did Not Protect from OA-like Cartilage Degeneration

**Figure 1:**
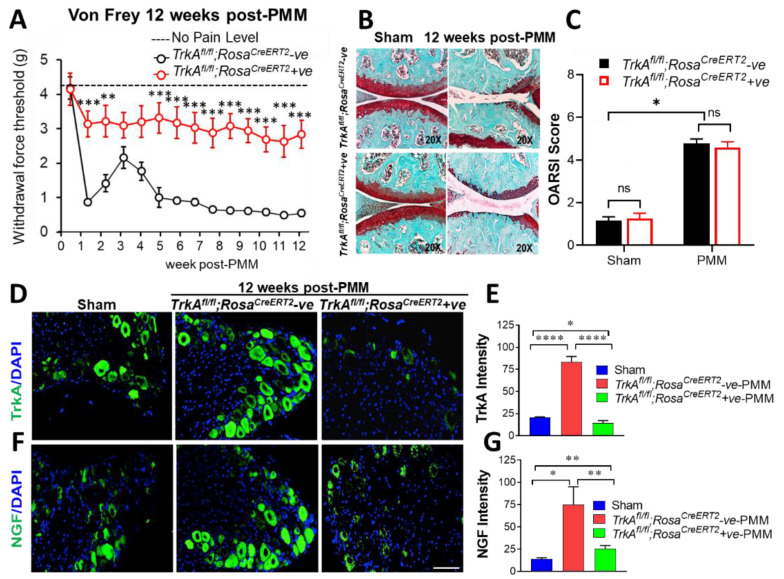
Global deletion of *TrkA* in mice relieved OA-associated hypersensitivity to pain and reduced expression of TrkA and NGF in the DRG but did not improve knee joint pathology with OA progression. (**A**) Development of mechanical allodynia (von Frey filament testing) in the ipsilateral hind paw, comparing *TrkA^fl/fl^;Rosa^CreERT2^*-negative (*n* = 11) and *TrkA^fl/fl^;Rosa^CreERT2^*-positive (*n* = 5) mice following PMM. (**B**) Histological assessment for proteoglycan depletion by Safranin-O fast green staining (×20). Each knee shown represents a group of mice (*n* = 4). (**C**) Severity of articular cartilage degradation was graded using the Osteoarthritis Research Society International (OARSI) scoring system. Values are mean ± SEM. Global deletion of *TrkA* in mice resulted in reduced expression of NGF. Immunofluorescence images of TrkA (**D**) and NGF (**F**) in the lumbar DRG (L3-L5) harvested at 12 weeks after PMM showed significant reduction of both TrkA and NGF in *TrkA^fl/fl^;Rosa^CreERT2^*-positive mice. The quantification of TrkA intensity (**E**) and NGF intensity (**G**) showed deletion of *TrkA* significantly reduced NGF in DRG (**F**,**G**). Values are mean ± SEM. * *p* < 0.05, ** *p* < 0.01, *** *p* < 0.001, **** *p* < 0.0001, ns: not significant. *p* = 0.742 (sham), *p* = 0.582 (PMM). Scale bar: 100 μm.

Upregulation of the NGF/TrkA axis exacerbates the OA-associated pain in synovium and in innervating dorsal root ganglion (DRG) sensory neurons, according to our previous studies with *PKCδ* null mice [11]. In order to elucidate the role of the NGF/TrkA signaling pathway in OA pain transmission and cartilage preservation, we first examined the effect of global conditional deletion of *TrkA*, a cognate receptor of NGF, by global tamoxifen-inducible ablation of the gene in *TrkA^fl/fl^;Rosa^CreERT2^* mice with five consecutive days of intraperitoneal (i.p) injection of tamoxifen at 4 weeks of age. Pain sensitivity and histopathological changes in the knee joint following OA induction by PMM surgery were analyzed. Pain tests measuring mechanical allodynia using von Frey filaments and other pain-associated behavior including increased frequency of grooming activity (data not shown) indicated that global deletion of TrkA in mice (*TrkA^fl/fl^;Rosa^CreERT2^* positive) shows insensitivity to OA pain compared to Cre-negative control mice that develop OA-associated hyperalgesia measured by withdrawal force thresholds (WFT, *p* < 0.01) (Figure 1A). Five additional pain tests, namely, the (i) ambulation test, (ii) rearing test, (iii) hot plate test, (iv) acetone test, and (v) burrowing test, were performed and are presented in Figure S1. The ambulation tests, which measure horizontal counts of beam interruptions when the animal moves about (Figure S1A), and the rearing test, which measures vertical counts of beam interruptions when the animal raises its posture (Figure S2) showed no meaningful differences. However, these two tests indicate that mice display normal motor function necessary to maintain posture and balance. In hot plate tests, both *TrkA^fl/fl^;Rosa^CreERT2^*-positive and -negative group mice showed sensitivity to heat after 4 weeks after PMM (Figure S1C). The acetone tests, which reflect sensitivity to low temperature, showed cold sensitivity in 4 weeks after PMM in *TrkA^fl/fl^;Rosa^CreERT2^*-positive mice (*p* < 0.01), while *TrkA^fl/fl^;Rosa^CreERT2^*-negative mice displayed significant sensitivity in 8 weeks after PMM (*p* < 0.01, Figure S1D). The burrowing tests, which measure the ability of animals to empty a tube filled with food pellets, did not display any meaningful differences in either *TrkA^fl/fl^;Rosa^CreERT2^*-positive mice or -negative mice (Figure S1E).

Despite the differences in the degree of pain sensitivity between the global deletion of *TrkA* mice (Cre positive) and *TrkA^fl/fl^;Rosa^CreERT2^*-negative littermates, these two groups showed no statistically significant difference in the development of OA-like pathological progression, as observed in Safranin-O fast green staining (Figure 1B) and histopathology grading by OARSI score (Figure 1C). Immunofluorescence staining validated over 95% deletion of *TrkA* in DRG, as shown in Figure 1D,E. Collectively, these results suggest that the NGF/TrkA signaling pathway is critically involved in developing OA pain sensation. Furthermore, our study also reaffirms our findings indicating that the severity of joint pain sensation does not always correlate with the degree of joint pathology [11].

### 2.2. Global Conditional Deletion of TrkA Resulted in Reduced Expression of TrkA and NGF in the DRG Sensory Neurons in Mice

OA was induced by PMM in 12-week-old *TrkA^fl/fl^;Rosa^CreERT2^*-positive mice (*n* = 5) and their litter mate controls, *TrkA^fl/fl^;Rosa^CreERT2^*-negative mice (*n* = 11). Immunofluorescence staining of TrkA and NGF in the lumbar L3-L5 DRG isolated at 12 weeks after PMM surgery were monitored. The expression levels of TrkA were significantly (*p* < 0.00001) reduced in *TrkA^fl/fl^;Rosa^CreERT2^*-positive mice in the lumbar DRG (Figure 1D) over 95%, as shown by quantification of TrkA expression per area (Figure 1E). Deletion of *TrkA* significantly (*p* < 0.013) reduced the expression of NGF (Figure 1F) by over 80%, as shown in the quantification of NGF expression per area (Figure 1G). These results are consistent with the concept that OA-induced hyperalgesia is associated with increased NGF/TrkA signaling.

### 2.3. Cartilage-Specific Deletion of TrkA Did Not Improve OA-Associated Hypersensitivity to Pain or Knee Joint Pathology with OA Progression in Mice

**Figure 2:**
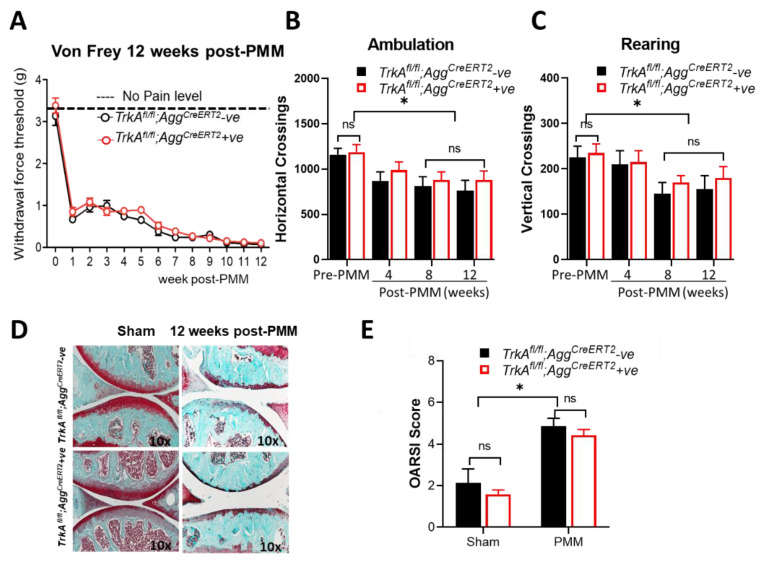
Cartilage-specific deletion of *TrkA* in mice did not improve either OA-associated hypersensitivity to pain or knee joint pathology with OA progression. OA was induced by PMM in 12-week-old *TrkA^fl/fl^;Aggrecan^CreERT2^*-positive (*n* = 13) and *TrkA^fl/fl^;Aggrecan^CreERT2^*-negative control mice (*n* = 6), followed by harvesting knee joints at 12 weeks after PMM. Each knee shown is representative of a group of mice. (**A**) Development of mechanical allodynia (von Frey filament testing) in the ipsilateral hind paw, comparing *TrkA^fl/fl^;Aggrecan^CreERT2^*-negative and *TrkA^fl/fl^;Aggrecan^CreERT2^*-positive mice following PMM. There were no significant differences. (**B**) Ambulation (horizontal photo beam crossings) and (**C**) spontaneous rearing activity (vertical photo beam crossings) were not different in *TrkA*-deleted mice compared with the control after PMM, while both activities were reduced between pre- and advanced OA. (**D**) Histological assessment for proteoglycan depletion by Safranin-O fast green staining (×10). (**E**) Severity of articular cartilage degradation was graded using the Osteoarthritis Research Society International (OARSI) scoring system. Values are mean ± SEM. * *p* < 0.05, ns: not significant. *p* = 0.644 (sham), *p* = 0.376 (PMM).

OA was induced by PMM in 12-week-old *TrkA^fl/fl^;Aggrecan^CreERT2^*-positive mice (*n* = 13) and their littermate controls, *TrkA^fl/fl^;Aggrecan^CreERT2^*-negative mice (*n* = 6), followed by harvesting of knee joints at 12 weeks after PMM surgery. Development of mechanical allodynia by von Frey filament testing in the ipsilateral hind paw was compared with *TrkA^fl/fl^;Aggrecan^CreERT2^*-negative mice and *TrkA^fl/fl^;Aggrecan^CreERT2^*-positive mice every week until 12 weeks after PMM surgery. Mice with a cartilage-specific deletion of *TrkA* did not exhibit significant differences in pain sensation (Figure 2A) and other pain-related behavior such as ambulation test measured by horizontal photo beam crossings (Figure 2B) and spontaneous rearing activity measured by vertical photo beam crossings (Figure 2C). Both ambulation and rearing activities were reduced between pre-PMM and post-PMM mice (Figure 2B,C). In hot plate tests (Figure S2A), both *TrkA^fl/fl^;Aggrecan^CreERT2^*-positive and -negative control group mice showed significant (*p* < 0.001) sensitivity to heat after 8 weeks after PMM, but there was no differences between cKO mice or negative control mice. In the acetone tests, *TrkA^fl/fl^;Aggrecan^CreERT2^*-negative mice displayed significant cold sensitivity in 12 weeks after PMM (*p* < 0.05), while *TrkA^fl/fl^;Aggrecan^CreERT2^*-positive mice displayed no significant sensitivity compared with pre-PMM or cKO mice (Figure S2B).

Cartilage-specific deletion of *TrkA* also did not alter proteoglycan depletion, as established by histological assessment with Safranin-O fast green staining (Figure 2D) nor the severity of articular cartilage degradation as determined using the Osteoarthritis Research Society International (OARSI) scoring system (Figure 2E). These results suggest that loss of NGF/TrkA signaling in cartilage does not affect catabolic pathways that perturb the cartilage extracellular matrix, nor does it affect the progression of OA hyperalgesia and cartilage deterioration.

### 2.4. Intra-Thecal Injection of Anti-NGF Antibody Showed No Effect on Joint Pain, While Intra-Articular Injection of Anti-NGF Antibody Improved OA-Induced Chronic Pain

**Figure 3:**
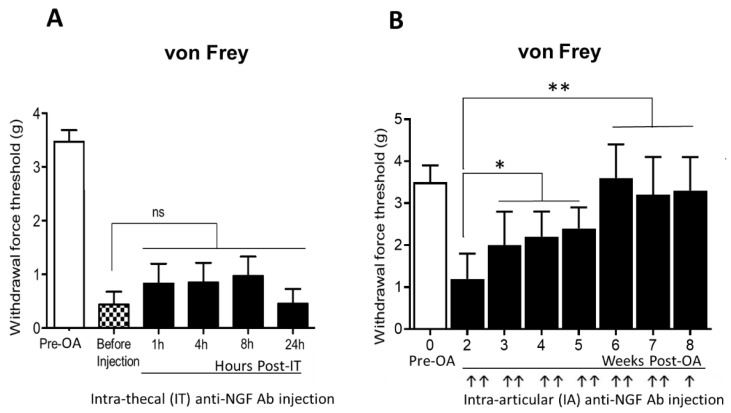
NGF/TrkA supported OA pain transmission in the peripheral but not in the central nervous system. (**A**) Intrathecal (IT) injection of anti-NGF antibody in C57BL/6 mice at 13 weeks after PMM did not alleviate hyperalgesia from chronic osteoarthritis, indicating that pain transmission is not a central event but may occur through peripheral sensory neurons. OA was induced by partial medial meniscectomy (PMM) in 12-week-old wild-type C57Bl/6 mice (*n* = 7), followed by pain test with von Frey filament. At 13 weeks after PMM, 10 μg of anti-NGF antibody in 5 μL (2 μg/μL) was injected intrathecally, and pain was assessed at 1 h, 4 h, 8 h, and 24 h after IT injection. (**B**) Von Frey filament testing in the ipsilateral hindpaw of PKCδ null mice (*n* = 10) receiving intaarticular (IA) injection of anti-NGF-2.5S antibody (30 μg in 5 μL saline) twice a week until 8 weeks after DMM surgery. Mice injected with anti-NGF antibody showed significantly reduced pain from the third week of anti-NGF IA injection. Black arrows indicate the IA injection. Adapted from Kc et al., Figure 6H [11]. * *p* < 0.05, ** *p* < 0.01, ns: not significant. *p* = 0.35.

Next, we tested anti-NGF antibody therapy to relieve pain and examined whether the action of NGF in pain transmission was peripheral. When an anti-NGF antibody was injected intra-thecally (IT) in C57Bl/6 mice at 13 weeks after PMM surgery, there was no improvement in pain reduction from chronic osteoarthritis (Figure 3A). However, when the anti-NGF antibody was delivered locally by intra-articular (IA) injection, the OA pain was significantly reduced (Figure 3B). IA injection of anti-NGF antibody was given twice a week to OA-induced PKCδ null mice by DMM, and significant pain reduction is shown in Figure 6H of Kc et al. [11]. These results indicate that pain is transmitted via peripheral sensory neurons and that peripheral inhibition of NGF/TrkA signaling can be effective. Hence, local inhibition of pain in OA can significantly reduce side effects, which may result from the whole-body application.

### 2.5. Sensory Neuron-Specific Deletion of TrkA (TrkA^fl/fl^;Na_V_1.8^CreERT2^-Positive) Abolished OA-Associated Hypersensitivity to Pain but Did Not Improve Knee Joint Pathology with OA Progression in Mice

To understand the function of NGF/TrkA signaling in peripheral sensory neurons, we selectively and conditionally deleted *TrkA* using Cre recombination under control of the promoter for voltage-gated sodium channel (Na_V_1.8/SCN10A). *TrkA^fl/fl^;Na_V_1.8^CreERT2^* mice were generated by crossing *TrkA^fl/fl^* mice with *Na_V_1.8^CreERT2^*, and conditional ablation was induced by systemic administration of 2 mg tamoxifen for 5 consecutive days starting at 4 weeks of age. OA was induced by PMM in 12-week-old *TrkA^fl/fl^;Na_V_1.8^CreERT2^*-positive mice and their corresponding littermate controls (*TrkA^fl/fl^;Na_V_1.8^CreERT2^*-negative mice), followed by pain testing every week and harvesting knee joints, DRG, and spinal cords (SCs) at 12 weeks after PMM surgery.

**Figure 4:**
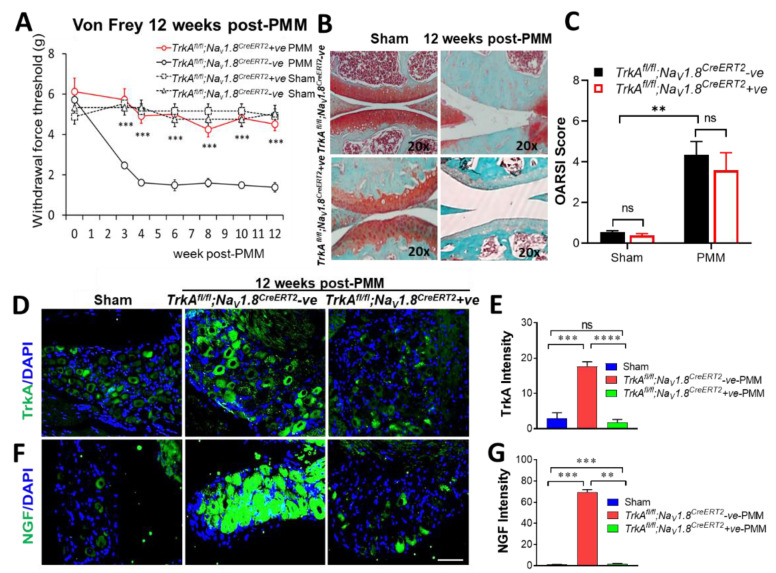
Sensory-neuron-specific deletion of *TrkA* in mice (*TrkA^fl/fl^;Na_V_1.8^CreERT2^*-positive) abolished OA-associated hypersensitivity to pain as effectively as global deletion of *TrkA*, but did not improve knee joint pathology with OA progression. (**A**) Development of mechanical allodynia (von Frey filament testing) in the ipsilateral hind paw, comparing *TrkA^fl/fl^;Na_V_1.8^CreERT2^*-negative (*n* = 9) and *TrkA^fl/fl^;Na_V_1.8^CreERT2^*-positive (*n* = 12) mice following PMM. (**B**) Histological assessment for proteoglycan content by Safranin-O fast green staining (×20). Each knee shown is representative of a group of mice (*n* = 12, 9, 7, 8). (**C**) Severity of articular cartilage degradation was monitored by the OARSI scoring (*n* = 5). (**D**) Sensory-neuron-specific deletion of TrkA in mice (*TrkA^fl/fl^;Na_V_1.8^CreERT2^*-positive) reduced NGF in DRG. Each image shown is representative of a group of mice (*n* = 5). Representative immunofluorescence staining for TrkA (green) in the DRG of *TrkA^fl/fl^;Na_V_1.8^CreERT2^*-positive and Cre- control *TrkA^fl/fl^;Na_V_1.8^CreERT2^*-negative mice. (**E**) Quantitative analysis of TrkA expression in the DRG. (**F**) Immunofluorescence staining for NGF (green) in DRG. (**G**) Quantitative analysis of NGF expression in DRG. All quantitative analysis values are mean ± SEM. ** *p* < 0.01, *** *p* < 0.001, **** *p* < 0.0001, ns: not significant. *p* = 0.284 (sham), *p* = 0.12 (PMM), *p* = 0.327 (sham vs. *TrkA^fl/fl^;Na_V_1.8^CreERT2^*-positive PMM). Scale bar: 100 μm.

Development of mechanical allodynia was measured by von Frey filament testing in the ipsilateral hind paw every week, and the pain response was compared in *TrkA^fl/fl^;Na_V_1.8^CreERT2^*-negative and *TrkA^fl/fl^;Na_V_1.8^CreERT2^*-positive mice following PMM or sham surgery. Sensory-neuron-specific deletion of *TrkA* in mice (*TrkA^fl/fl^;Na_V_1.8^CreERT2^*-positive) abolished OA-associated hypersensitivity (Figure 4A). This effect is comparable to global deletion of *TrkA* in mice (*TrkA^fl/fl^;Rosa^CreERT2^*-positive), as shown in Figure 1A. However, proteoglycan content as visualized by Safranin-O fast green staining (×20) showed that the joint continued to deteriorate upon OA-induced damage in mice with sensory-neuron-specific deletion of *TrkA*. Hence, loss of *TrkA* did not significantly improve cartilage structure protection compared to mice in the *TrkA^fl/fl^;Na_V_1.8^CreERT2^*-negative control group (Figure 4B). Severity of articular cartilage degradation was graded using the Osteoarthritis Research Society International (OARSI) scoring system to quantitate the results of cartilage protection. The OARSI scores further confirmed no significant difference in cartilage protection against OA-induced damage with sensory neuron-specific ablation of *TrkA* (Figure 4C).

### 2.6. Characterization of Sensory-Neuron-Specific Deletion of TrkA in Mice (TrkA^fl/fl^;Na_V_1.8^CreERT2^-Positive Mice)

Immunofluorescence staining for TrkA in the DRG of *TrkA*-deleted mice and control mice (Figure 4D), as well as the quantitative analysis of TrkA expression in the DRG (Figure 4E) indicate that TrkA protein expression in DRG is significantly reduced (over 90%, *p* < 0.000001), reflecting the efficiency of sensory neuron-specific deletion of *TrkA* in *TrkA^fl/fl^;Na_V_1.8^CreERT2^*-positive mice. TrkA intensity measured in ImageJ software from the National Institutes of Health (NIH imagej.nih.gov/ij/download.html: accessed on 8 July, 2020) significantly increased sixfold from a baseline of 2.96 to 17.71 upon OA induction (*p* < 0.0002 in sham vs. *TrkA^fl/fl^;Na_V_1.8^CreERT2^*-negative PMM mice). However, TrkA intensity decreased to 1.82 upon sensory-neuron-specific loss of *TrkA* (*p* < 0.00001 in *TrkA^fl/fl^;Na_V_1.8^CreERT2^*-negative PMM vs. *TrkA^fl/fl^;Na_V_1.8^CreERT2^*-positive PMM) (Figure 4E). These immunofluorescence results of DRG establish that *TrkA* is effectively deleted by tamoxifen-induced Cre activation in sensory neurons of *TrkA^fl/fl^;Na_V_1.8^CreERT2^* mice (Figure 4D,E).

### 2.7. Sensory Neuron-Specific Deletion of TrkA in Mice (TrkA^fl/fl^;Na_V_1.8^CreERT2^-Positive) Reduced NGF Significantly

NGF expression was significantly reduced in *TrkA^fl/fl^;Na_V_1.8^CreERT2^*-positive mice on the basis of immunofluorescence staining (Figure 4F) and quantitative analysis of NGF protein in DRG (Figure 4G). NGF intensity increased dramatically (69-fold) from a baseline of 1.01 to 69.30 in *TrkA^fl/fl^;Na_V_1.8^CreERT2^*-negative OA mice (*p* < 0.00001 in sham vs. *TrkA^fl/fl^;Na_V_1.8^CreERT2^*-negative PMM). However, levels of NGF expression return to close to the control level 1.92 (*p* < 0.00001 in *TrkA^fl/fl^;Na_V_1.8^CreERT2^*-negative PMM vs. *TrkA^fl/fl^;Na_V_1.8^CreERT2^*-positive PMM) (Figure 4F,G). These results clearly indicate that OA-induced hyperalgesia is associated with increased NGF/TrkA axis signaling in DRG.

### 2.8. Decreased Density of Peripheral Nerve Fibers in OA Synovium Correlated with Decreased Joint Pain Sensation

**Figure 5:**
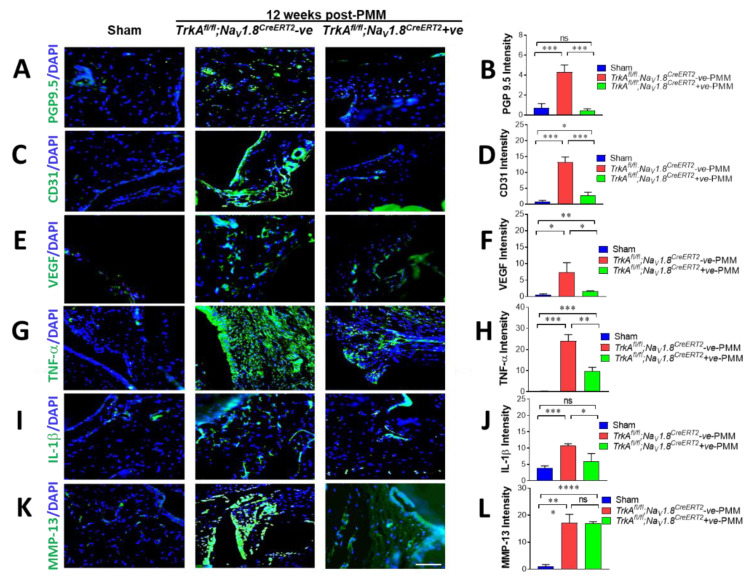
Immunofluorescence staining of peripheral nerve fiber sprouting, angiogenesis factor, pro-inflammatory cytokines, and MMP-13 in osteoarthritis (OA) synovium correlated with decreased joint pain sensation in OA. Reduced PGP9.5 (**A**,**B**) expression in joint tissues indicated less synovial sensory fiber and downregulated neural fiber. (**A**) Representative immunofluorescence images of staining for PGP9.5 (green) in knee joint synovium of *TrkA^fl/fl^;Na_V_1.8^CreERT2^*-negative (*n* = 9) and *TrkA^fl/fl^;Na_V_1.8^CreERT2^*-positive (*n* = 12) mice. (**B**) Quantitative analyses of nerve fiber sprouting. The density of PGP9.5 fibers of *TrkA^fl/fl^;Na_V_1.8^CreERT2^*-negative mice exhibited a significant increase in synovial/capsular regions compared to *TrkA^fl/fl^;Na_V_1.8^CreERT2^*-positive mice. PGP9.5+ nerve fiber density was calculated as the intensity of the nerve fiber area. 4′,6-Diamidino-2-phenylindole (DAPI) stained nuclei blue. *** *p* < 0.001. (**C**–**F**) Reduced angiogenesis factor CD31 (**C**) and VEGF (**E**) and quantification of CD31 (**D**) and VEGF (**F**) expression in joint tissues indicated decreased innervation and angiogenesis markers. Significantly decreased expression of both CD31 and VEGF (green; (**C**,**E**)) in the knee joint synovium of *TrkA*-deleted mice after PMM compared to *TrkA^fl/fl^;Na_V_1.8^CreERT2^*-negative control mice. Each group: *n* = 5. Quantitative analysis of CD31 (**D**) and VEGF (**F**) expression in the synovium. (**G**–**L**) Pro-inflammatory cytokines TNF-α, IL-1β, and cartilage-degrading enzyme MMP-13 correlated with the unprotected joint pathology. Representative immunofluorescence images of tumor necrosis factor-α (TNF-α) (green) expression in the synovium of *TrkA^fl/fl^;Na_V_1.8^CreERT2^*-positive mice and *TrkA^fl/fl^;Na_V_1.8^CreERT2^*-negative control mice are shown in (**G**). Each group: *n* = 5. Quantitative analyses of TNF-α expression in cartilage and synovium (**H**). (**I**) Representative immunofluorescence images of interleukin (IL)-1β expression (green) in cartilage and synovium of *TrkA^fl/fl^;Na_V_1.8^CreERT2^*-positive mice and *TrkA^fl/fl^;Na_V_1.8^CreERT2^*-negative control mice are shown. Each group: *n* = 5. (**J**) Quantitative analyses of IL-1β expression in cartilage and synovium. (**K**) Immunofluorescence images of MMP-13 expression in the synovium of *TrkA^fl/fl^;Na_V_1.8^CreERT2^*-positive and *TrkA^fl/fl^;Na_V_1.8^CreERT2^*-negative mice. Each group: *n* = 5. (**L**) Quantitative analyses of MMP-13 expression in synovium. Values are mean ± SEM. * *p* < 0.05, ** *p* < 0.01, *** *p* < 0.001, **** *p* < 0.0001, ns: not significant. *p* = 0.418 (PGP 9.5 sham vs. *TrkA^fl/fl^;Na_V_1.8^CreERT2^*-positive PMM), *p* = 0.230 (IL-1β sham vs. *TrkA^fl/fl^;Na_V_1.8^CreERT2^*-positive PMM), *p* = 0.911 (MMP-13 *TrkA^fl/fl^;Na_V_1.8^CreERT2^*-negative vs.-positive PMM). 4′,6-Diamidino-2-phenylindole (DAPI) stained nuclei blue. Scale bar: 50 μm.

**Figure 6:**
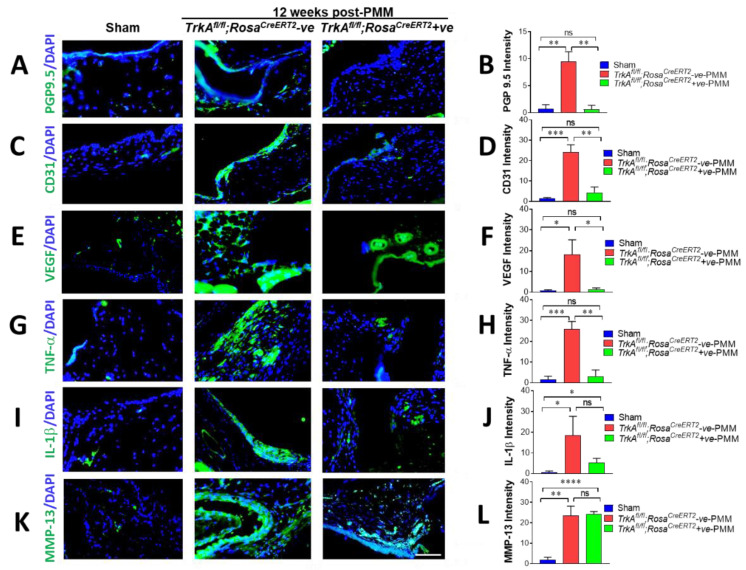
Immunofluorescence staining of peripheral nerve fiber sprouting, angiogenesis factor, pro-inflammatory cytokines, and MMP-13 in osteoarthritis (OA) synovium correlated with decreased joint pain sensation in OA. Reduced PGP9.5 expression in joint tissues indicated less synovial sensory fiber and downregulated neural fiber. (**A**) Representative immunofluorescence images of staining for PGP9.5 (green) in knee joint synovium of *TrkA^fl/fl^;Rosa^CreERT2^*-negative (*n* = 11) and *TrkA^fl/fl^;Rosa^CreERT2^*-positive (*n* = 5) mice. (**B**) Quantitative analyses of nerve fiber sprouting. PGP9.5+ nerve fiber density was calculated as the intensity of the nerve fiber area. 4′,6-Diamidino-2-phenylindole (DAPI) stained nuclei blue. (**C**–**F**) Reduced angiogenesis factor CD31 (**C**) and VEGF (**E**) and quantification of CD31 (**D**) and VEGF (**F**) expression in joint tissues indicated decreased innervation and angiogenic markers in the knee joint synovium of *TrkA*-deleted mice after PMM compared to *TrkA^fl/fl^;Rosa^CreERT2^*-negative control mice. Quantitative analysis of CD31 (**D**) VEGF (**F**) expression in the synovium. Immunofluorescence staining of TNF-α (**G**) and interleukin (IL)-1β (**I**) expression of MMP-13 (**K**) in *TrkA^fl/fl^;Rosa^CreERT2^*-positive mice following PMM surgery. Representative immunofluorescence images of tumor necrosis factor-α (TNF-α) (green) expression in synovium of *TrkA^fl/fl^;Rosa^CreERT2^*-positive mice and *TrkA^fl/fl^;Rosa^CreERT2^*-negative control mice are shown in (**G**). Each group: *n* = 5. Quantitative analyses of TNF-α expression in the synovium (**H**). (**I**) Representative immunofluorescence images of interleukin (IL)-1β expression (green) in cartilage and synovium of *TrkA^fl/fl^;Rosa^CreERT2^*-positive mice and *TrkA^fl/fl^;Rosa^CreERT2^*-negative control mice are shown. Each group: *n* = 5. (**J**) Quantitative analyses of IL-1β expression in cartilage and synovium. (**K**) Immunofluorescence images of MMP-13 expression in the synovium of *TrkA^fl/fl^;Rosa^CreERT2^*-positive and *TrkA^fl/fl^; Rosa^CreERT2^*-negative mice. Each group: *n* = 5. (**L**) Quantitative analyses of MMP-13 expression in the synovium. Values are mean ± SEM. * *p* < 0.05, ** *p* < 0.01, *** *p* < 0.001, **** *p* < 0.0001, ns: not significant. *p* = 0.867 (PGP 9.5 sham vs. *TrkA^fl/fl^;Rosa^CreERT2^*-positive PMM), *p* = 0.138 (CD31 sham vs. *TrkA^fl/fl^;Rosa^CreERT2^*-positive PMM), *p* = 0.390 (VEGF sham vs. *TrkA^fl/fl^;Rosa^CreERT2^*-positive PMM), *p* = 0.464 (TNF-α sham vs. *TrkA^fl/fl^;Rosa^CreERT2^*-positive PMM), *p* = 0.073 (IL-1β *TrkA^fl/fl^Rosa^CreERT2^*-negative vs. -positive PMM), *p* = 0.986 (MMP-13 *TrkA^fl/fl^;Rosa^CreERT2^*-negative vs. -positive PMM). 4′,6-Diamidino-2-phenylindole (DAPI) stained nuclei blue. Scale bar: 50 μm.

We next investigated whether sensory nerve sprouting in the knee joints of mice may contribute to hyperalgesia as a principal pathological feature of OA. Peripheral nerve innervation increased in the ipsilateral knee joint synovium at 12 weeks after PMM surgery, as shown by immunofluorescence staining with anti-protein gene product 9.5 (PGP 9.5) antibody (Figure 5A and Figure 6A). The results show that induction of OA by PMM surgery significantly increased the density of PGP9.5-positive nerve fibers in synovial/capsular regions of *TrkA^fl/fl^;Na_V_1.8^CreERT2^*-negative and *TrkA^fl/fl^;Rosa^CreERT2^*-negative mice compared to sham control mice. PGP9.5-positive nerve fiber density was calculated as the signal intensity per nerve fiber surface area, as reflected by the number of nuclei stained with 4′,6-diamidino-2-phenylindole (DAPI) blue. PGP9.5-positive structures (green) in the knee joint synovium were significantly reduced upon loss of *TrkA* in both *TrkA^fl/fl^;Na_V_1.8^CreERT2^*-positive and *TrkA^fl/fl^;Rosa^CreERT2^*-positive mice compared with both negative control *TrkA^fl/fl^;Na_V_1.8^CreERT2^*-negative and *TrkA^fl/fl^;Rosa^CreERT2^*-negative mice (Figure 5A and Figure 6A), and this was confirmed in the quantification of the intensity of PGP 9.5 (Figure 5B and Figure 6B). PGP9.5 in sham-operated control mice were 0.69 and 0.74, and they are significantly increased 6-fold and 13-fold in intensity to 4.30 and 9.47 in *TrkA^fl/fl^;Na_V_1.8^CreERT2^*-negative and *TrkA^fl/fl^;Rosa^CreERT2^*-negative PMM induced OA mice, respectively (*p* < 0.0018 and *p* < 0.0016 in sham vs. *TrkA^fl/fl^;Na_V_1.8^CreERT2^*-negative and *TrkA^fl/fl^;Rosa^CreERT2^*-negative PMM mice, respectively). However, PGP9.5 expression levels in OA induced mice were completely reduced to 0.43 and 0.63 in *TrkA^fl/fl^;Na_V_1.8^CreERT2^*-positive and *TrkA^fl/fl^;Rosa^CreERT2^*-positive OA mice, respectively (*p* < 0.0008 and *p* < 0.0014 in *TrkA^fl/fl^;Na_V_1.8^CreERT2^*-negative and *TrkA^fl/fl^;Rosa^CreERT2^*-negative PMM vs. *TrkA^fl/fl^;Na_V_1.8^CreERT2^*-positive and *TrkA^fl/fl^;Rosa^CreERT2^*-positive PMM, respectively) (Figure 5A,B and Figure 6A,B).

These results indicate that the degree of OA pain correlated with increased peripheral nerve fibers in knee joints of OA mice and deletion of *TrkA* in the peripheral sensory neurons. Hence, inactivation of NGF/TrkA signaling can effectively block OA-associated pain transmission.

### 2.9. OA-Induced Angiogenesis in Joint Synovium Was Mitigated by SensoryNeuron-Specific Deletion of TrkA Mice

The endothelium marker CD31, which is a potent angiogenesis factor (Figure 5C,D and Figure 6C,D), was monitored to understand OA-mediated angiogenesis after loss of *TrkA* in sensory neurons. Immunofluorescence staining for CD31 in the synovial vasculature of the ipsilateral knee joint of 12 weeks after PMM surgery showed no obvious aggressive angiogenesis in *TrkA^fl/fl^;Na_V_1.8^CreERT2^*-positive mice and *TrkA^fl/fl^;Rosa^CreERT2^*-positive mice compared to *TrkA^fl/fl^;Na_V_1.8^CreERT2^* negative and *TrkA^fl/fl^;Rosa^CreERT2^* negative PMM mice. CD31 expression increased strongly (18-fold) in the ipsilateral knee joint of both *TrkA^fl/fl^;Na_V_1.8^CreERT2^*-negative mice and *TrkA^fl/fl^;Rosa^CreERT2^*-negative mice at 12 weeks after PMM surgery, as shown by immunofluorescence staining with anti-CD31 antibody (Figure 5C and Figure 6C). However, the fluorescence intensity of CD31 (green) in knee joints was significantly reduced upon loss of *TrkA* in both of *TrkA^fl/fl^;Na_V_1.8^CreERT2^*-positive mice and *TrkA^fl/fl^;Rosa^CreERT2^*-positive mice comparing both of *TrkA^fl/fl^;Na_V_1.8^CreERT2^*-negative and *TrkA^fl/fl^;Rosa^CreERT2^*-negative mice (Figure 5D and Figure 6D). CD31 intensities in sham-operated control mice were 0.73 and 1.32 and were significantly increased to 13.24 upon induction of OA in *TrkA^fl/fl^;Na_V_1.8^CreERT2^*-negative mice (*p* < 0.0002) and to 24.26 in *TrkA^fl/fl^;Rosa^CreERT2^*-negative PMM mice (*p* < 0.0004), respectively. This elevation of CD31 signals was severely reduced to intensity to 2.66 in *TrkA^fl/fl^;Na_V_1.8^CreERT2^*-positive mice (*p* < 0.0007) and 4.32 in *TrkA^fl/fl^;Rosa^CreERT2^*-positive PMM mice (*p* < 0.0015) compared to their respective negative control PMM mice (Figure 5C,D, and Figure 6C,D).

Consistent with these findings, VEGF expression was strongly increased (14-fold and 20-fold, respectively) in the ipsilateral knee joint of *TrkA^fl/fl^;Na_V_1.8 ^CreERT2^*-negative and *TrkA^fl/fl^;Rosa^CreERT2^*-negative mice upon induction of OA at 12 weeks after PMM surgery, as measured by immunofluorescence signals for anti-VEGF antibody (Figure 5E and Figure 6E). However, VEGF expression in the knee joint synovium was significantly reduced upon loss of *TrkA* in two different genetic mouse models comparing both *TrkA^fl/fl^;Na_V_1.8^CreERT2^*-positive and *TrkA^fl/fl^;Rosa^CreERT2^*-positive mice with both *TrkA^fl/fl^;Na_V_1.8^CreERT2^*-negative mice and *TrkA^fl/fl^;Rosa^CreERT2^*-negative mice (Figure 5F and Figure 6F). As expected, VEGF intensities were significantly increased by OA induction, comparing 0.53 and 0.90 in sham-operated control mice to 7.42 with *TrkA^fl/fl^;Na_V_1.8^CreERT2^*-negative PMM mice (*p* < 0.015) and 18.18 with *TrkA^fl/fl^;Rosa^CreERT2^*-negative PMM mice (*p* < 0.013), respectively. However, this increase in VEGF levels was strongly mitigated (85% and 97%) by loss of *TrkA* to intensity 1.59 (*p* < 0.025) in *TrkA^fl/fl^;Na_V_1.8^CreERT2^*-positive OA mice and 1.36 (*p* < 0.015) in *TrkA^fl/fl^;Rosa^CreERT2^*-positive PMM mice (Figure 5E,F and Figure 6E,F).

Collectively, our results indicate that NGF/TrkA signaling in sensory nerves is important for angiogenesis and that inhibition of this pathway may alleviate both OA-enhanced angiogenesis and pain sensation.

### 2.10. SensoryNeuron-Specific Deletion of TrkA Mitigated Pro-Inflammatory Cytokines

We next measured tumor necrosis factor-α (TNF-α/TNF) and interleukin-1β (IL-1β/IL1B) expression, the most common pro-inflammatory cytokines in OA pathology. To identify the tissue source of inflammatory cytokine production, we examined expression levels of TNF-α and IL-1β. Protein levels of TNF-α (Figure 5G,H and Figure 6G,H) and IL-1β (Figure 5I,J and Figure 6I,J) significantly increased in cartilage and synovium at 12 weeks after PMM (comparing *TrkA^fl/fl^;Na_V_1.8^CreERT2^*-negative and *TrkA^fl/fl^;Rosa^CreERT2^*-negative mice with sham control mice). However, the OA-induced elevation of these cytokines was impeded substantially upon *TrkA* loss in *TrkA^fl/fl^;Na_V_1.8^CreERT2^*-positive and *TrkA^fl/fl^;Rosa^CreERT2^*-positive mice.

TNF-α expression was over 16-fold increased in the ipsilateral knee joint of both *TrkA^fl/fl^;Na_V_1.8 ^CreERT2^*-negative and *TrkA^fl/fl^;Rosa^CreERT2^*-negative mice at 12 weeks after PMM surgery, as shown in immunofluorescence staining (Figure 5G and Figure 6G). However, TNF-α expression (green fluorescence) was 60% reduced in knee joints of *TrkA^fl/fl^;Na_V_1.8 ^CreERT2^*-positive mice, while *TrkA^fl/fl^;Rosa^CreERT2^*-positive mice exhibited 93% reduction compared to their control PMM mice (Figure 5G and Figure 6G), and this was confirmed in the quantification of the density of TNF-α (Figure 5H and Figure 6H). TNF-α intensity in sham-operated control mice was 0.22, and it was greatly increased to an intensity of 24.14 in *TrkA^fl/fl^;Na_V_1.8^CreERT2^*-negative OA mice (*p* < 0.0001) and the intensity increased from 1.6 to 25.8 in *TrkA^fl/fl^;Rosa^CreERT2^*-negative PMM mice (*p* < 0.0005). However, expression of TNF-α in OA-induced mice was reduced to 9.84 in *TrkA^fl/fl^;Na_V_1.8^CreERT2^*-positive mice (*p* < 0.0018) and 3.21 in *TrkA^fl/fl^;Rosa^CreERT2^*-positive mice (*p* < 0.001) (Figure 5H and Figure 6H).

IL-1β expression was increased 2.7-fold in the ipsilateral knee joint of *TrkA^fl/fl^;Na_V_1.8^CreERT2^*-negative mice and increased 34.7-fold in *TrkA^fl/fl^;Rosa^CreERT2^*-negative mice at 12 weeks after PMM surgery, as shown by immunofluorescence staining with anti-IL-1β antibody (Figure 5I and Figure 6I). However, green fluorescence indicating IL-1β expression in knee joints was reduced 70% in *TrkA^fl/fl^;Na_V_1.8^CreERT2^*-positive mice, while *TrkA^fl/fl^;Rosa^CreERT2^*-positive mice showed 74% reduction in fluorescence intensity (Figure 5J and Figure 6J). IL-1β intensity in sham-operated control mice was 3.96, and it was significantly increased to an intensity of 10.73 in *TrkA^fl/fl^;Na_V_1.8^CreERT2^*-negative OA mice (*p* < 0.00017) and increased from 0.53 to 18.5 in *TrkA^fl/fl^;Rosa^CreERT2^*-negative mice (*p* < 0.028). The expression decreased to an intensity of 5.97 in *TrkA^fl/fl^;Na_V_1.8^CreERT2^*-positive PMM mice (*p* < 0.029) and 5.29 in *TrkA^fl/fl^;Rosa^CreERT2^*-positive OA-induced mice (*p* = 0.073) (Figure 5J and Figure 6J).

Although *TrkA*-deleted OA mice showed substantial reductions in the expression of pro-inflammatory cytokines, this inhibition was only partial because pro-inflammatory cytokine activities still remained in the knee joints of PMM-induced *TrkA* cKO mice and facilitated the destruction of joint integrity. This result may explain why loss of *TrkA* greatly relieves pain transmission (see Figure 4A) but does not provide noticeable protection in joint pathology shown (see Figure 4B,C).

### 2.11. Cartilage-Degrading Enzyme MMP-13 Remained Activated upon SensoryNeuron-Specific Deletion of TrkA

The expression of cartilage-degrading enzyme MMP-13/MMP13, a critical target in OA progression, was not eliminated in OA-induced *TrkA^fl/fl^;Na_V_1.8^CreERT2^*-positive mice or *TrkA^fl/fl^;Rosa^CreERT2^*-positive mice (Figure 5K and Figure 6K) at all.

MMP-13 expression was increased 16-fold in the ipsilateral knee joint of *TrkA^fl/fl^;Na_V_1.8 ^CreERT2^*-negative mice and increased 22-fold in *TrkA^fl/fl^;Rosa^CreERT2^*-negative mice at 12 weeks after PMM surgery, compared to the sham control, as shown by immunofluorescence staining (Figure 5K and Figure 6K). However, loss of *TrkA* did not reduce MMP-13 level in OA-induced *TrkA* deleted mice (*TrkA^fl/fl^;Na_V_1.8^CreERT2^*-positive and *TrkA^fl/fl^;Rosa^CreERT2^*-positive mice compared with their negative control mice), as confirmed in the intensity quantification (Figure 5L and Figure 6L). MMP-13 level in sham-operated mice was 1.12, and it was increased 16-fold (intensity 17.2) in OA-induced *TrkA^fl/fl^;Na_V_1.8^CreERT2^*-negative mice (*p* < 0.001) and increased from 1.09 to 23.5 in *TrkA^fl/fl^;Rosa^CreERT2^*-negative mice (*p* < 0.001). However, expression of MMP-13 in OA-induced *TrkA* cKO mice remained high in both sensory-neuron-specific deletion (17.0, *p* = 0.91) and in global deletion (23.5, *p* = 0.99) of *TrkA* (Figure 5L and Figure 6L).

Immunofluorescence of MMP-13 showed NGF/TrkA is not a critical mediator in joint destruction in OA. Deletion of *TrkA*, and thus inhibition of NGF signaling specifically at the sodium channel sensory neuron (Na_V_1.8), fails to eliminate MMP-13, a cartilage-degrading enzyme in PMM-induced OA mice. This finding indicates that TrkA/NGF signaling impacts pain transmission but not the joint pathology, at least in part, because it has no effect on the MMP-13 activity in mediating cartilage destruction.

### 2.12. SensoryNeuron-Specific and Global Deletion of TrkA Reduced SP and CGRP Significantly in the DRG of OA-Induced Mice

Even though the degree of cartilage degeneration was similar in both *TrkA^fl/fl^;Na_V_1.8^CreERT2^*-positive and *TrkA^fl/fl^;Na_V_1.8^CreERT2^*-negative mice (Figure 4B,C), there was significant pain relief in *TrkA^fl/fl^;Na_V_1.8^CreERT2^*-positive PMM mice (Figure 4A). Therefore, we compared the expression levels of pain transmitter substance P (SP) and calcitonin-gene-related peptide (CGRP) in DRG.

**Figure 7:**
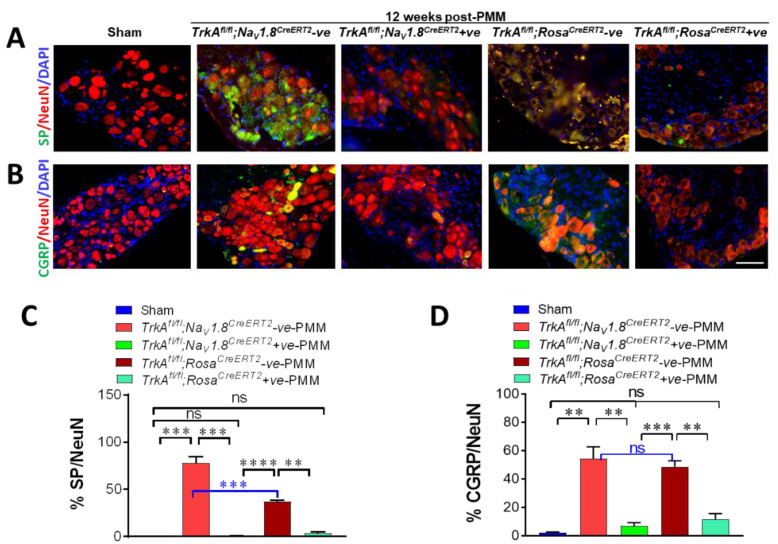
Sensory-neuron-specific deletion of *TrkA* in OA mice (*TrkA^fl/fl^;Na_V_1.8^CreERT2^*-positive) and global deletion of *TrkA* in OA mice (*TrkA^fl/fl^;Rosa^CreERT2^*-positive) reduced pain molecules substance P and CGRP significantly in DRG. (**A**) Representative immunofluorescence staining for SP (green), NeuN (red), and 4′,6-diamidino-2-phenylindole (DAPI)-stained nuclei (blue) in the DRG of sham, *TrkA^fl/fl^;Na_V_1.8^CreERT2^*-negative PMM mice, *TrkA^fl/fl^;Na_V_1.8^CreERT2^*-positive PMM mice, *TrkA^fl/fl^;Rosa^CreERT2^*-negative OA mice, and *TrkA^fl/fl^;Rosa^CreERT2^*-positive OA mice. (**B**) Representative immunofluorescence staining for CGRP (green), NeuN (red), and 4′,6-diamidino-2-phenylindole (DAPI)-stained nuclei (blue) in the DRG of sham, *TrkA^fl/fl^;Na_V_1.8^CreERT2^*-negative PMM mice, *TrkA^fl/fl^;Na_V_1.8^CreERT2^*-positive PMM mice, *TrkA^fl/fl^;Rosa^CreERT2^*-negative PMM mice, and *TrkA^fl/fl^;Rosa^CreERT2^*-positive PMM mice. (**C**) Quantitative analysis of SP expression in the DRG. (**D**) Quantitative analysis of CGRP expression in the DRG. Values are mean ± SEM. ** *p* < 0.01, *** *p* < 0.001, **** *p* < 0.0001, ns: not significant. *p* = 0.117 (SP sham vs. *TrkA^fl/fl^;Rosa^CreERT2^*-positive PMM), *p* = 0.06 (SP sham vs. *TrkA^fl/fl^;Na_V_1.8^CreERT2^*-positive PMM), *p* = 0.08 (CGRP sham vs. *TrkA^fl/fl^;Rosa^CreERT2^*-positive PMM), *p* = 0.596 (CGRP *TrkA^fl/fl^;Na_V_1.8^CreERT2^*-negative PMM vs. *TrkA^fl/fl^;Rosa^CreERT2^*-negative PMM). Scale bar: 100 μm.

Immunofluorescence staining for substance P (SP: green) co-stained with the neuronal marker NeuN (red) in DRG (Figure 7A) and their quantitative analysis of SP expression in the NeuN-positive cells in DRG (Figure 7C) exhibited significant reduction in both *TrkA^fl/fl^;Na_V_1.8^CreERT2^*-positive mice and *TrkA^fl/fl^;Rosa^CreERT2^*-positive OA mice (*p* < 0.003). SP intensity in sham control mice was 0.43 and was significantly increased to 78 in *TrkA^fl/fl^;Na_V_1.8^CreERT2^*-negative PMM mice (*p* < 0.0003) and 37.25 in *TrkA^fl/fl^;Rosa^CreERT2^*-negative OA mice (*p* < 0.00005). However, loss of *TrkA* resulted in 99% reduction of SP expression to control level 0.80 in *TrkA^fl/fl^;Na_V_1.8^CreERT2^*-positive OA mice (*p* < 0.00030) and 77% reduction to 8.65 in *TrkA^fl/fl^;Rosa^CreERT2^*-positive OA mice (*p* < 0.004) (Figure 7A,C).

Likewise, CGRP expression (green) visualized with NeuN (red) co-staining showed 91% reduction in *TrkA^fl/fl^;Na_V_1.8^CreERT2^*-positive mice (*p* < 0.006) and 79% reduction in *TrkA^fl/fl^;Rosa^CreERT2^*-positive OA mice (*p* < 0.003) compared to their Cre negative littermate control OA mice. CGRP intensity in sham mice was 2.09, and it was significantly increased to 54.24 in *TrkA^fl/fl^;Na_V_1.8^CreERT2^*-negative OA mice (*p* < 0.003) and 48.75 in *TrkA^fl/fl^;Rosa^CreERT2^*-negative OA mice (*p* < 0.014). Over 90% reduction of CGRP expression in both *TrkA* cKO mice returned the level down to 6.91 in *TrkA^fl/fl^;Na_V_1.8^CreERT2^*-positive OA mice (*p* < 0.01) and 11.66 in *TrkA^fl/fl^;Rosa^CreERT2^*-positive OA mice (*p* < 0.01) (Figure 7B,D).

This indicates that the deletion of *TrkA* in sensory neurons significantly reduces pain molecules SP and CGRP in DRG. SP and CGRP are also known as angiogenesis neuropeptides and it is suggested that the NGF/TrkA signaling in sensory nerve has significant effects on angiogenesis for alleviating pain sensation and OA-induced hyperalgesia.

### 2.13. SensoryNeuron-Specific Deletion of TrkA in Mice (TrkA^fl/fl^;Na_V_1.8^CreERT2^-Positive) Reduced Glial Cell Activity, Inflammatory Cytokines, and CGRP Significantly in Spinal Cords

**Figure 8:**
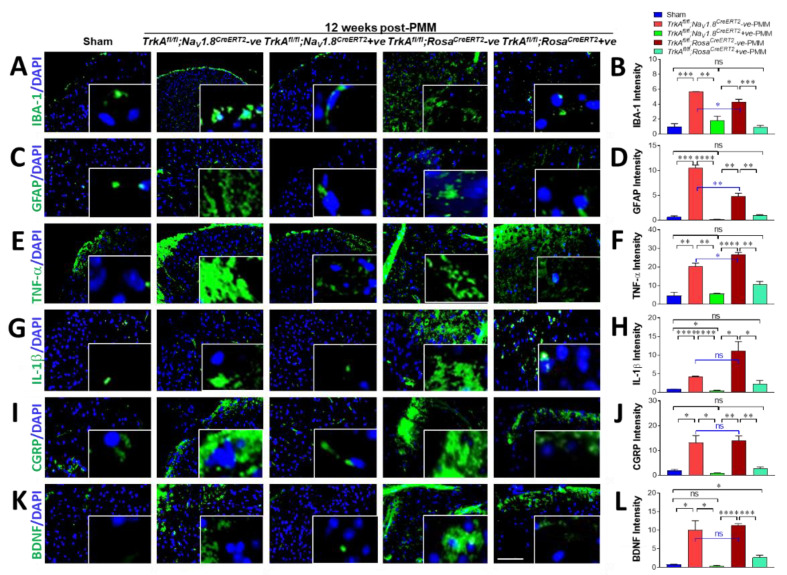
Sensory-neuron-specific deletion of *TrkA* in mice (*TrkA^fl/fl^;Na_V_1.8^CreERT2^*-positive) reduced glial cell activity and pain molecule CGRP and BDNF significantly in spinal cords. (**A**) Representative immunofluorescence images of staining for IBA-1 (green) in SC of *TrkA^fl/fl^;Na_V_1.8^CreERT2^*-negative (*n* = 9), *TrkA^fl/fl^;Na_V_1.8^CreERT2^*-positive (*n* = 12), *TrkA^fl/fl^;Rosa^CreERT2^*-negative (*n* = 11), and *TrkA^fl/fl^;Rosa^CreERT2^*-positive (*n* = 5) mice. (**B**) Quantitative analyses of IBA-1. Following PMM surgery, 4′,6-diamidino-2-phenylindole (DAPI) stained nuclei blue. (**C**,**D**) Astroglial marker GFAP expression (green; (**C**)) and quantification of GFAP (**D**) in the SC of *TrkA*-deleted mice after PMM compared to *TrkA^fl/fl^;Na_V_1.8^CreERT2^*-negative (*n* = 9), *TrkA^fl/fl^;Na_V_1.8^CreERT2^*-positive (*n* = 12), *TrkA^fl/fl^;Rosa^CreERT2^*-negative (*n* = 11), and *TrkA^fl/fl^;Rosa^CreERT2^*-positive (*n* = 5) mice. 4′,6-Diamidino-2-phenylindole (DAPI) stained nuclei blue. Not significant compared between sham control and *TrkA^fl/fl^;Na_V_1.8^CreERT2^* +ve mice and *TrkA^fl/fl^;Rosa^CreERT2^*-positive with PMM. Representative immunofluorescence images of tumor necrosis factor-α (TNF-α) expression and quantitative analyses (**E**,**F**), interleukin (IL)-1β expression and quantitative analyses (**G**,**H**), CGRP expression and quantitative analyses (**I**,**J**), and BDNF expression and quantitative analyses (**K**,**L**) in SC of sham control, *TrkA^fl/fl^;Na_V_1.8^CreERT2^*-negative, *TrkA^fl/fl^;Na_V_1.8^CreERT2^* +ve, *TrkA^fl/fl^;Rosa^CreERT2^*-negative, and *TrkA^fl/fl^;Rosa^CreERT2^*-positive mice are shown. Values are mean ± SEM. 4′,6-Diamidino-2-phenylindole (DAPI) stained nuclei blue. * *p* < 0.05, ** *p* < 0.01 *** *p* < 0.001, **** *p* < 0.0001, ns: not significant. *p* = 0.962 (IBA-1 sham vs. *TrkA^fl/fl^;Rosa^CreERT2^*-positive PMM), *p* = 0.062 (TNF-α sham vs. *TrkA^fl/fl^;Rosa^CreERT2^*-negative PMM), *p* = 0.234 (IL-1β sham vs. *TrkA^fl/fl^;Rosa^CreERT2^*-positive PMM), *p* = 0.051 (IL-1β *TrkA^fl/fl^;Na_V_1.8^CreERT2^*-negative PMM vs. *TrkA^fl/fl^;Rosa^CreERT2^*-negative PMM), *p* = 0.434 (CGRP sham vs. *TrkA^fl/fl^;Rosa^CreERT2^*-positive PMM), *p* = 0.809 (CGRP *TrkA^fl/fl^;Na_V_1.8^CreERT2^*-negative PMM vs. *TrkA^fl/fl^;Rosa^CreERT2^*-negative PMM), *p* = 0.123 (BDNF sham vs. *TrkA^fl/fl^ Na_V_1.8^CreERT2^*-positive PMM), *p* = 0.663 (BDNF *TrkA^fl/fl^;Na_V_1.8^CreERT2^* vs. *TrkA^fl/fl^Rosa^CreERT2^*-negative PMM). Scale bar: 50 μm.

In order to determine whether the mechanism involved in the improvement of hyperalgesia by peripheral sensory neuronal inhibition of NGF/TrkA signaling was based on retrograde centralized pain transportation, we examined glial cell activity, inflammatory cytokines, and pain molecules in the spinal cords of OA-induced mice. The microglial cell marker IBA-1 in OA-induced *TrkA^fl/fl^;Na_V_1.8^CreERT2^*-negative mice was increased sixfold in spinal cords (*p* < 0.0004) and fourfold in *TrkA^fl/fl^;Rosa^CreERT2^*-negative OA mice (*p* < 0.0031) but was reduced by 83% in OA-induced *TrkA^fl/fl^;Na_V_1.8^CreERT2^*-positive mice (*p* < 0.0029) and 100% in *TrkA^fl/fl^;Rosa^CreERT2^*-positive OA mice (*p* < 0.0009). Quantitative analyses of IBA-1 was calculated as the intensity of IBA-1(green) area (Figure 8A,B). This indicated the increased microglial cell activity due to OA induction was greatly reduced in *TrkA* cKO mice in that NGF and pain molecule retrograde transportation was diminished by deletion of *TrkA* in sensory neurons.

The astroglial cell marker GFAP in OA-induced *TrkA^fl/fl^;Na_V_1.8^CreERT2^*-negative mice was increased 16-fold and in *TrkA^fl/fl^;Rosa^CreERT2^*-positive OA mice in spinal cords compared to sham control mice, which increased 8-fold (*p* < 0.0001 and *p* < 0.0025, respectively). However, loss of *TrkA* was reduced completely in OA-induced *TrkA^fl/fl^;Na_V_1.8^CreERT2^*-positive mice (*p* < 0.00008) and by 93% in *TrkA^fl/fl^;Rosa^CreERT2^*-positive OA mice (*p* < 0.003) (Figure 8C,D).

This finding indicated that astroglial cell activation due to OA induction was abolished in *TrkA^fl/fl^;Na_V_1.8^CreERT2^*-positive mice and drastically reduced in *TrkA^fl/fl^;Rosa^CreERT2^*-positive OA mice. This change in astroglial activation was due to deletion of TrkA in sensory neurons, which impedes retrograde transportation of augmented NGF levels upon OA induction. Hence, astroglial activity to produce inflammatory cytokines and pain molecules is significantly abolished. Interestingly, complete reduction of astroglial cell activity is achieved in sensory-neuron-specific deletion in that NGF and pain molecule retrograde transportation is solely accountable to deletion of *TrkA* in sensory neurons.

Next, we investigated tumor necrosis factor-α (TNF-α) and interleukin-1β (IL-1β) expression in SCs to see whether OA-induced glial cell activity produced inflammatory cytokines. Expression levels of TNF-α (Figure 8E,F) and IL-1β (Figure 8G,H) significantly increased in spinal cords of *TrkA^fl/fl^;Na_V_1.8^CreERT2^*-negative and *TrkA^fl/fl^;Rosa^CreERT2^*-negative OA mice at 12 weeks after PMM compared to sham control mice. TNF-α expression was increased fourfold in SC of OA-induced *TrkA^fl/fl^;Na_V_1.8^CreERT2^*-negative mice and increased sixfold in *TrkA^fl/fl^;Rosa^CreERT2^*-negative mice compared to the sham surgery mice (*p* < 0.0041 and *p* < 0.0005, respectively). However, TNF-α expression in SC was 93% reduced in *TrkA^fl/fl^;Na_V_1.8^CreERT2^*-positive mice (*p* < 0.0016) and 72% reduced in *TrkA^fl/fl^;Rosa^CreERT2^*-positive OA mice (*p* < 0.001). TNF-α intensity in sham-operated control mice was 4.6 and greatly increased to 20.3 upon OA-induction in *TrkA^fl/fl^;Na_V_1.8^CreERT2^*-negative mice (*p* < 0.0041) and 26.8 in *TrkA^fl/fl^;Rosa^CreERT2^*-negative mice (*p* < 0.0005). However, TNF-α expression in OA-induced *TrkA* cKO mice was significantly reduced to 5.7 in *TrkA^fl/fl^;Na_V_1.8^CreERT2^*-positive OA mice (*p* < 0.0016) and 10.8 in *TrkA^fl/fl^;Rosa^CreERT2^*-positive OA mice (*p* < 0.001) (Figure 8E,F).

IL-1β expression was increased 5-fold in the SC of *TrkA^fl/fl^;Na_V_1.8^CreERT2^*-negative mice and increased 12-fold in *TrkA^fl/fl^;Rosa^CreERT2^*-positive OA mice at 12 weeks after PMM surgery, as shown by immunofluorescence staining with anti-IL-1β antibody (Figure 8G). However, IL-1β expression in SC was completely abolished in *TrkA^fl/fl^;Na_V_1.8^CreERT2^*-positive mice (*p* < 0.00002) and 87% reduced in *TrkA^fl/fl^;Rosa^CreERT2^*-positive OA mice (*p* < 0.03) (Figure 8G,H). IL-1β intensity in sham control mice was 0.9 and was significantly increased to an intensity of 4.3 in OA-induced *TrkA^fl/fl^;Na_V_1.8^CreERT2^*-negative mice (*p* < 0.000003) and 11.37 in *TrkA^fl/fl^;Rosa^CreERT2^*-positive OA mice (*p* < 0.015). A total of 100% reduction occurred at intensity 0.5 in *TrkA^fl/fl^;Na_V_1.8^CreERT2^*-positive OA mice (*p* < 0.00002) and a 87% reduction in 2.24 in *TrkA^fl/fl^;Rosa^CreERT2^*-positive OA mice (*p* < 0.03) (Figure 8G,H).

Even though sensory-neuron-specific deletion of *TrkA* exhibited partial reduction of pro-inflammatory cytokines in the knee joints of OA-induced *TrkA^fl/fl^;Na_V_1.8^CreERT2^*-positive mice, expression of TNF-α and IL-1β was reduced by over 90% in spinal cords. This indicated that glial cell activity due to retrograde pain transportation was significantly reduced by the sensory neuron-specific deletion of *TrkA*.

Loss of *TrkA* completely inhibited CGRP expression in the SC of OA-induced *TrkA^fl/fl^;Na_V_1.8^CreERT2^*-positive mice and *TrkA^fl/fl^;Rosa^CreERT2^*-positive mice (Figure 8I,J) compared to their negative control PMM mice. CGRP intensity in sham-operated mice 2.0 had a 6.5-fold increase to 13.2 in OA-induced *TrkA^fl/fl^;Na_V_1.8^CreERT2^*-negative mice (*p* < 0.017) and a 7-fold increase to 14.1 in OA-induced *TrkA^fl/fl^;Rosa^CreERT2^*-negative mice (*p* < 0.003). However, expression of CGRP in PMM-induced mice was reduced to 0.86 in *TrkA^fl/fl^;Na_V_1.8^CreERT2^*-positive PMM mice (*p* < 0.012) and to 2.73 in *TrkA^fl/fl^;Rosa^CreERT2^*-positive OA mice (*p* < 0.004), indicating complete inhibition of glial activity (Figure 8I,J) was achieved in sensory-neuron-specific *TrkA*-deleted mice.

### 2.14. SensoryNeuron-Specific Deletion of TrkA in Mice (TrkA^fl/fl^;Na_V_1.8^CreERT2^-Positive) Abolished the Central Pain Mediator, Brain Derived Neurotrophic Factor (BDNF), in Spinal Cords

Finally, we investigated whether increased peripheral sensory NGF due to OA stimulated brain derived neurotrophic factor (BDNF), a key pain mediator in the central nervous system (CNS), and whether BDNF is diminished upon sensory-neuron-specific deletion of *TrkA*, hence inhibiting the NGF/TrkA signaling pathway.

Immunofluorescence staining of BDNF in *TrkA^fl/fl^;Na_V_1.8^CreERT2^*-positive mice and *TrkA^fl/fl^;Rosa^CreERT2^*-positive mice following PMM surgery (Figure 8K,L) showed complete inhibition of BDNF expression in SCs compared to control PMM mice. BDNF intensity in sham control mice was 0.79, and it was increased 13-fold to 10.13 in *TrkA^fl/fl^;Na_V_1.8^CreERT2^*-negative OA mice (*p* < 0.019) and increased 14-fold to 11.29 in *TrkA^fl/fl^;Rosa^CreERT2^*-negative OA mice (*p* < 0.00003). However, expression of BDNF was reduced to 0.41 in *TrkA^fl/fl^;Na_V_1.8^CreERT2^*-positive PMM mice (*p* < 0.016) and to 2.67 in *TrkA^fl/fl^;Rosa^CreERT2^*-positive OA mice (*p* < 0.0004). This showed a complete inhibition of the central pain mediator BDNF (Figure 8K,L) was accomplished by sensory-neuron-specific *TrkA* deletion, thus disabling the NGF/TrkA signaling pathway.

## 3. Discussion

OA is a disease of the whole joint, and OA pain is associated with the presence of bone marrow lesions and synovitis [26]. Our previous studies have shown that ablation of the *PKCδ*/Prkcd gene prevents cartilage destruction and yet exacerbates OA-associated pain [11]. Pain perception during progression of OA may not strictly correlate with the degree of cartilage damage because neurons and blood vessels are absent in mature articular cartilage.

Here, we present evidence that the NGF/TrkA pathway is critical for transducing pain and changes in nerve processing, as well as the fact that both peripheral and central sensitization may contribute to pain in OA. Augmentation of NGF/TrkA signaling in the joint synovium and the peripheral level of sensory neurons are the major determinants that facilitate pro-nociception and the transition of OA from an asymptomatic joint degenerative process to a painful disease. However, inhibiting the NGF/TrkA pathway to desensitize OA-associated hyperalgesia does not improve cartilage preservation, consistent with the concept that OA pain may develop independently of joint destruction.

Many growth factors and cytokines, including NGF, bFGF, VEGF, and IL-1, are regulated by autocrine/paracrine feedback mechanisms. We observed that blocking VEGFR1/Flt1 or VEGF2/Flk1 significantly downregulated VEGF expression (Ma et al., unpublished data). We previously observed that augmentation of NGF caused increased expression of TrkA, which is the cognate receptor for NGF [11]. In our current study, increased NGF transportation to DRG was impaired due to sensory-neuron-specific deletion of NGF receptor TrkA. Therefore, NGF was downregulated after TrkA was deleted in the sensory neurons.

In knee joints, NGF is produced by fibroblast-like synoviocytes and/or chondrocytes under OA conditions. Yet, cartilage-specific deletion of *TrkA* (as the cognate NGF receptor) fails to protect from OA-associated hyperalgesia. This finding suggests that NGF signaling in cartilage may not be catabolic or that *TrkA* deletion in cartilage is not sufficient to block the progression of OA. In contrast, deletion of *TrkA* in sensory neurons modulated the activity of NGF, angiogenesis, and pro-inflammatory cytokines in the knee joint of mice upon OA induction after PMM surgery. More importantly, mice experienced remarkable pain relief during early to advanced stages of OA in our study. The protection from OA-induced hyperalgesia upon sensory-neuron-specific deletion of *TrkA* is directly supported by our observations that loss of *TrkA* decreased peripheral nerve fibers in knee joints and reduced angiogenesis.

Expression of pro-inflammatory cytokines was partially reduced in the synovium but was completely abolished in the spinal cord. This result indicates that these pro-inflammatory cytokines in the synovium play important roles in joint integrity. The complete ablation of cytokine production observed in the spinal cord was the result of the inactivation of glial cells, which are able to produce these inflammatory factors when provoked. NGF may upregulate IL-1β through TrkA and NF-κB/NFKB1-dependent caspase-1/CASP1 activation in human monocytes [21]. Pro-inflammatory cytokines TNF-α and IL-1β were upregulated in cartilage and synovium in advanced human OA and upon surgical induction of OA in mice [11]. Ligand binding of NGF to TrkA activates transcriptional and posttranscriptional pathways that mediate IL-1β release [27], while IL-1β induces chondrocyte inflammation and osteoarthritis via the NF-κB signaling pathway [28]. Sensory neuron-specific deletion of *TrkA* in OA-induced mice did not fully deactivate pro-inflammatory cytokine production, in agreement with the idea that the levels of TNF-α and IL-1β correlate with joint pathology but not with the level of pain.

The matrix metalloprotease MMP-13/MMP13 is a major collagen-degrading enzyme responsible for cartilage destruction in OA, and its inhibition has been considered a strategy to block OA development [29]. Our study showed that MMP-13 was still fully active during OA progression in mice upon sensory-neuron-specific *TrkA* deletion or global *TrkA* deletion. Therefore, we propose that MMP-13 is a critical factor for joint pathology protection, but its expression is not completely dependent on the NGF/TrkA pathway.

Deletion of *TrkA* in sensory neurons significantly reduced expression of pain molecules such as SP and CGRP in DRG and spinal cords that also act as angiogenic neuropeptides. NGF/TrkA signaling in sensory neurons stimulated angiogenesis factors such as VEGF and CD31, and, as a consequence, contributed to angiogenesis. This result suggests that NGF/TrkA signaling in sensory nerves contributes significantly to angiogenesis while alleviating pain sensation and OA-induced hyperalgesia.

Activated glial cell activity in our OA mouse model may have involved retrograde centralized pain transportation as a key mechanism that improves hyperalgesia upon peripheral sensory neuronal inhibition of NGF/TrkA signaling. Both microglial and astroglial cell activities were modulated, indicating that hyperalgesia in both early and advanced OA can be treated by *TrkA* inactivation. OA induces expression of the brain-derived pain mediator BDNF by peripheral NGF, and this induction of BDNF was completely abolished upon sensory-neuron-specific deletion of *TrkA*, which inactivates the NGF/TrkA signaling pathway.

Our findings strongly support an emerging concept for the etiology of OA in that the severity of joint pain is due to pathological changes in the synovium as well as cellular and molecular plasticity in the sensory neurons of the innervating DRG. Our results also support the mechanistic interpretation that NGF/TrkA signaling is one of the key determinants for the painful symptomatic transition of OA. Peripheral inhibition of NGF/TrkA signaling can effectively reduce the pain mechanism that operates via retrograde centralized pain sensitization. Hence, sensory-neuron-specific deletion of *TrkA* is as efficient as a global deletion and is sufficient for alleviating pain. Therefore, disease intervention would benefit from local inactivation of *TrkA* to eliminate the risk and safety issues of systemic treatments (which are modulated in mouse OA by global deletion of *TrkA*). Beyond localized inhibition of TrkA, other interventions will be required for cartilage repair and protection in advanced OA.

Clinical studies with analgesics, including non-steroidal anti-inflammatory drugs (NSAIDs), opioid agonists, and calcitonin, have been evaluated for therapeutic benefits to treat chronic pain [30]. Our finding that intervention in NGF/TrkA signaling suppresses OA hyperalgesia has important clinical ramifications and could be developed further as a superb and safe therapy against chronic pain.

One study limitation is that our findings on NGF/TrkA signaling in nociceptive responses associated with OA were based on a post-traumatic surgical mouse model for OA that may not strictly correlate with the age-related degenerative human OA. However, our results can be further tested for human applications using human-derived sensory-neuron-like stem cells. Another limitation is that targeting NGF/TrkA signaling is effective only for the alleviation of nociception but not for preserving joint pathology. Therefore, future studies may require the intercalation of OA disease-modifying drugs that inhibit nociception and preserve joint integrity simultaneously. A new experimental model could include chondrocytic three-dimensional cultures with primary cells from human patients and mouse models in which NGF/TrkA signaling is modified using nanoparticles with controlled release. Immunofluorescence microscopy and Western blots are both semi-quantitative because immunofluorescence and chemiluminescence each have a limited dynamic range. We were not able to present additional assessments of NGF/TrkA in Western blotting due to the size limitation of DRG, where the NGF receptor TrkA is selectively expressed in nociceptive DRG neurons [31]. However, fluorescence signals are exquisitely sensitive and provide definitive information of the local presence of proteins in situ within histological slides. In our study, we used well-characterized antibodies that have been extensively used in the field to detect TrkA and NGF in mouse sensory neurons in the DRG in situ. Our study did not present cortex/hippocampus-related data since Na_V_1.8 is typically selectively expressed at high levels in sensory ganglion neurons but not within the CNS. Moreover, no loss of neuronal cells nor any differences in motor function or other abnormal behavior in tamoxifen-inducible Cre-ERT2 recombinase deletion has been reported [32]. The behavioral response to pain assessments between global deletion of TrkA and sensory-neuron-specific deletion of TrkA (Na_V_1.8 expressing neurons) are similar.

Our findings can be summarized as follows: (1) Conditional whole-body deletion of *TrkA* in mice abolished OA pain. (2) Sensory neuron-specific deletion of *TrkA* alleviated OA pain as effectively as whole-body deletion of *TrkA* in mice. (3) Cartilage-specific deletion of *TrkA* in mice led to development of joint pain and damage in OA, showing no improvement on OA progression. (4) Peripheral sensory neuron-specific deletion of *TrkA* modulated OA-induced sensory neuron plasticity and abolished OA pain by interfering with the NGF/TrkA signaling pathway of retrograde pain transmission. (5) Sensory neuronal *TrkA* deletion did not downregulate the cartilage-degrading enzyme, hence leading to cartilage degeneration progresses. (6) Peripheral inhibition of NGF/TrkA signaling can effectively reduce pain, but other interventions are needed for cartilage repair and protection. In conclusion, this study provides compelling evidence for the role of NGF/TrkA in OA-related pain perception and supports the idea that this pathway is a viable target for novel disease-modifying pharmacotherapies.

## 4. Materials and Methods

### 4.1. Experimental Animals

Experimental mice were generated by crossing TrkA floxed mice (*TrkA^fl/fl^*) with *Rosa^CreERT2^* mice from Jackson (008463: B6.129-Gt (ROSA)26Sortm1(cre/ERT2) Tyj/J) for tamoxifen-inducible *TrkA^fl/fl^;Rosa^CreERT2^* mice. For tamoxifen-inducible *TrkA^fl/fl^;Aggrecan^CreERT2^* mice, TrkA floxed mice were crossed with *Aggrecan^CreERT2^* mice (Jackson Laboratory 019,148–B6.Cg-Acantm1(cre/ERT2)Crm/J). Tamoxifen-inducible *TrkA^fl/fl^;Nav1.8^CreERT2^* mice were generated by mating TrkA floxed mice with *Nav1.8^CreERT2^* mice, obtained from John Wood’s lab.

Tamoxifen-inducible conditional deletion of *TrkA* was achieved globally with *TrkA^fl/fl^;Rosa^CreERT2^* mice, cartilage-specific deletion with *TrkA^fl/fl^;Aggrecan^CreERT2^* mice, and sensory neuron-specific deletion with *TrkA^fl/fl^;Nav1.8^CreERT2^* mice by intraperitoneal injection of tamoxifen (2 mg/day) for 5 consecutive days at 4 weeks of age. The dose was optimized and ensured for the safety of growing young mice bone health [33]. Our *Nav1.8-CreERT2* constructs were specific for sensory neurons, and the Na_V_1.8 driver does not support Cre recombination in the CNS [32,34,35]. Tamoxifen-independent recombination was not a major concern in our system, since less than 1% of neurons showed Cre expression in the absence of tamoxifen treatment [32].

Mice were housed under standard laboratory conditions (in a temperature-controlled (21 ± 1 °C) room with a normal 12 h light/12 h dark cycle). Animal studies were performed according to guidelines in the Guide for the Care and Use of Laboratory Animals of the National Institutes of Health. All animal procedures were approved by the Jessie Brown Veterans Affairs Medical Center’s Institutional Animal Care and Use Committee (IACUC, protocol # 18–13). Surgeries were performed under anesthesia, and all efforts were made to minimize pain and suffering.

### 4.2. Surgical Procedure

All the surgical operations were performed under a microscope in an aseptic setting. Mice were positioned supine and given anesthesia with 1.5% isoflurane (Abbott Laboratories, North Chicago, IL, USA) in oxygen via a facemask. Osteoarthritis was induced in mice by partial medial meniscectomy (PMM) by cutting the medial menisco-tibial ligament (MMTL) and removing the medial meniscus (MM). Briefly, MMTL was cut to dislodge MM from the tibia, and then a portion of MM of around 1mm was cut with a surgical knife.

We performed PMM on *TrkA^fl/fl^;Rosa^CreERT2^*-negative mice (*n* = 11), *TrkA^fl/fl^;Rosa^CreERT2^*-positive mice (*n* = 5), *TrkA^fl/fl^;Aggrecan^CreERT2^*-negative mice (*n* = 6), *TrkA^fl/fl^;Aggrecan^CreERT2^*-positive (*n* = 13) mice, sensory-neuron-specific *TrkA^fl/fl^;Nav1.8^CreERT2^*-negative (*n* = 9), and *TrkA^fl/fl^;Nav1.8^CreERT2^*-positive (*n* = 12) mice. Sham surgery was performed without PMM on five wild-type mice and on *TrkA^fl/fl^;Nav1.8^CreERT2^*-negative (*n* = 8) and *TrkA^fl/fl^;Nav1.8^CreERT2^*-positive (*n* = 7) mice.

### 4.3. Animal Pain Behavioral Tests

Longitudinal pain behavior assessments were performed weekly in PMM or sham control groups from weeks 1 to 12 after surgery.

Mechanical allodynia by von Frey testing: Allodynia was evaluated on the basis of hind paw withdrawal from mechanical stimuli [36,37]. After allowing mice to accommodate for 10 min on a wire mesh grid, a calibrated set of von Frey filaments were applied from below to the plantar hind paw to determine the 50% force withdrawal threshold using an iterative method. A brisk lifting of the foot was recorded as a positive response.

Activity monitor: Animals were tested in clean vivarium plastic cages (42 × 25 × 20 cm) enclosed in a cage rack Photobeam Activity System (San Diego Instruments, San Diego, CA, USA). One set of photobeams was placed at foot level above the cage floor (with adjacent beams 5 cm apart) to measure ambulation (horizontal counts of beam interruptions when the animal walked). Another set of photobeams was placed 5 cm above the cage floor to measure rearing (vertical counts of beam interruptions when the animal stood). Activity was monitored in a dark room for 30 min.

### 4.4. Histology

At 12 week after surgery, mice were euthanized via CO_2_, and knee joints, DRG, and lumbar spinal cords were harvested for histological analysis. Safranin-O fast green staining was performed using 0.1% Safranin-O solution and 0.05% fast green solution for histological assessment of tissue morphology. The degree of joint damage was graded using the OARSI scoring system [38].

### 4.5. Immunofluorescence Staining

Knee joints, the lumbar part of the spinal cords, and L3-5 DRG were harvested, fixed, and embedded. All samples were cut at 5 μm thickness. After removing paraffin, antigens were retrieved, permeabilized, and blocked. Sections were then incubated overnight with primary antibodies: anti TrkA (EP1058Y: Abcam 76291, Waltham, MA, USA), anti-rabbit tumor necrosis factor-alpha (anti-TNF-α) (1:200; Novus Biologicals, Littleton, CO, USA), anti-rabbit interleukin (IL)-1β (anti-IL-1β) (1:100; Abcam 283818, Waltham, MA, USA), anti-rabbit nerve growth factor (anti-NGF) (1:200; Santa Cruz Biotechnology, Santa Cruz, CA, USA), anti-CD31 (1:100; Abcam 182981, Waltham, MA, USA), anti-rabbit protein gene product 9.5 (anti-PGP9.5) (1:100; Abcam 8189, Waltham, MA, USA), anti-substance P (1:100; Abcam 14184, Waltham, MA, USA), anti-CGRP (1:100; Abcam 81887, Waltham, MA, USA), anti-MMP13 (1:100; Abcam 219620, Waltham, MA, USA), anti-BDNF (1:100; Thermo-Fisher PAI-18357, Waltham, MA, USA), and anti-NeuN (1:100; Abcam 177487, Waltham, MA, USA) primary antibodies, followed by incubation with Alexa 488 or Alexa 555 fluorescent antibody conjugate (1:250; Invitrogen, Carlsbad, CA, USA). Immunofluorescence was examined using a fluorescence microscope (Nikon Eclipse NiE, Nikon Instruments Inc., Melville, NY, USA).

### 4.6. Statistical Analysis

GraphPad Prism 8.02 (Graph pad Software, San Diego, CA, USA) software was used for statistical analyses. All data are presented as mean ± SEM. Pain data at each time point were normalized to those obtained pre-surgery and were analyzed using a general linear model for repeated measures. The differences between PMM and sham groups were assessed with Student’s *t*-test and analysis of variance (ANOVA), as well as a post hoc Tukey test. *p*  ≤  0.05 was considered significant.

## 5. Conclusions

We investigated the mechanisms of pain transmission via the ligand NGF and its cognate receptor TrkA by utilizing OA mice with conditional deletion of *TrkA* in the peripheral sensory neurons, cartilage-specific deletion, and global deletion of *TrkA*. Conditional deletion of *TrkA* in peripheral sensory neurons improved OA-associated hyperalgesia by suppressing (i) NGF production in fibroblast-like synoviocytes, (ii) synovial angiogenesis, (iii) pain molecules, and (iv) glial cell activation. However, the collagen-degrading enzyme MMP-13 was not reduced, and thus sensory-neuron-specific deletion of *TrkA* failed to prevent cartilage damage in the joints of OA mice.

## Data Availability

Not applicable.

## Supplementary Materials

The following supporting information can be downloaded at: https://www.mdpi.com/article/10.3390/ijms232012076/s1.
