# Supplementary figures and images for: Sensory Neuron-Specific Deletion of Tropomyosin Receptor Kinase A (TrkA) in Mice Abolishes Osteoarthritis (OA) Pain via NGF/TrkA Intervention of Peripheral Sensitization
Source: Int J Mol Sci. 2022 Oct 11;23(20):12076. doi: 10.3390/ijms232012076 (PMC9602682; doi:10.3390/ijms232012076)

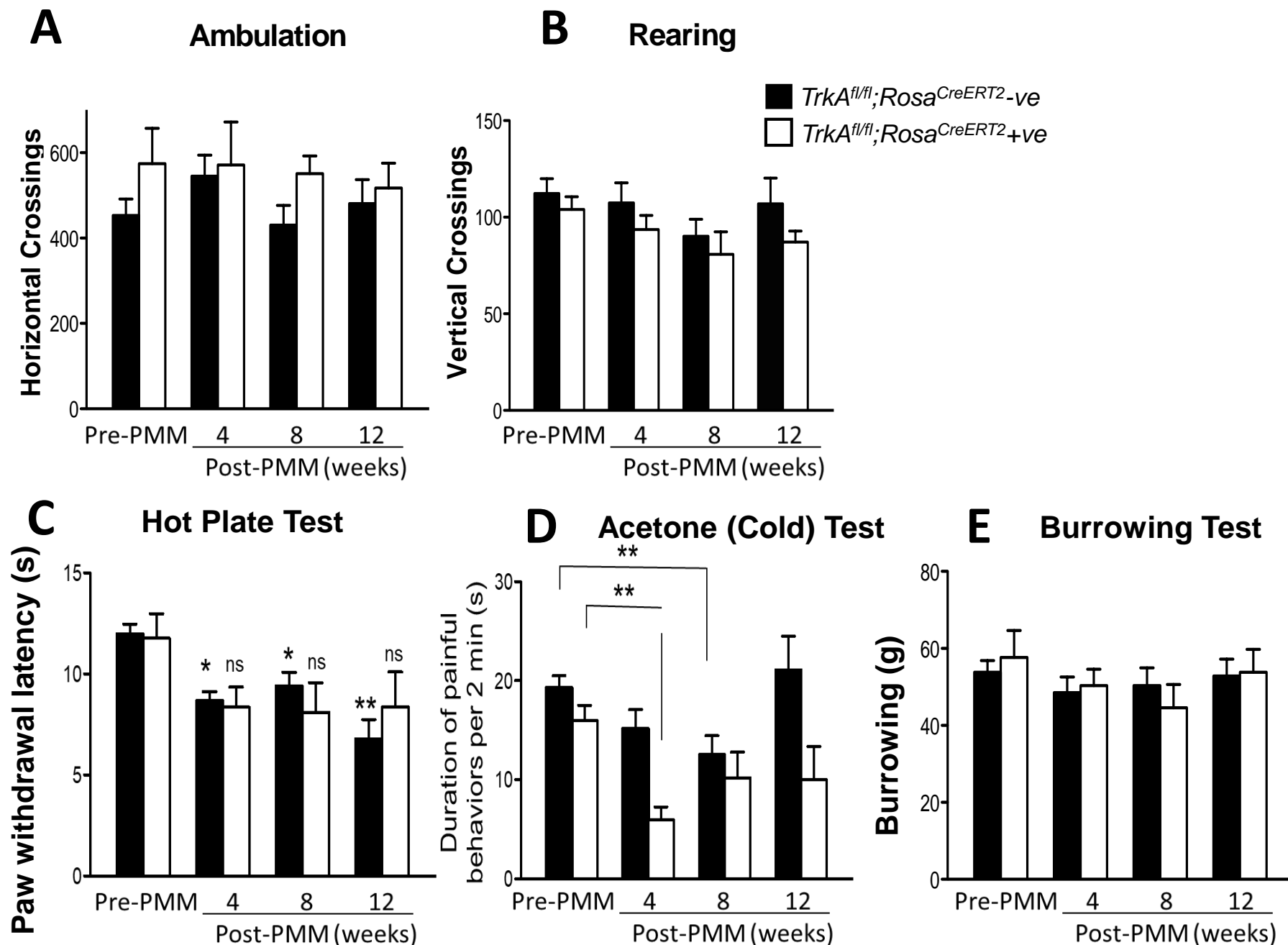

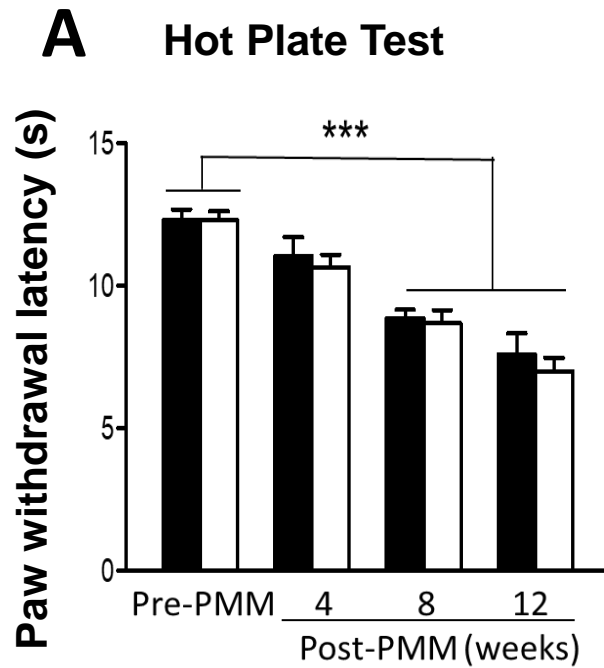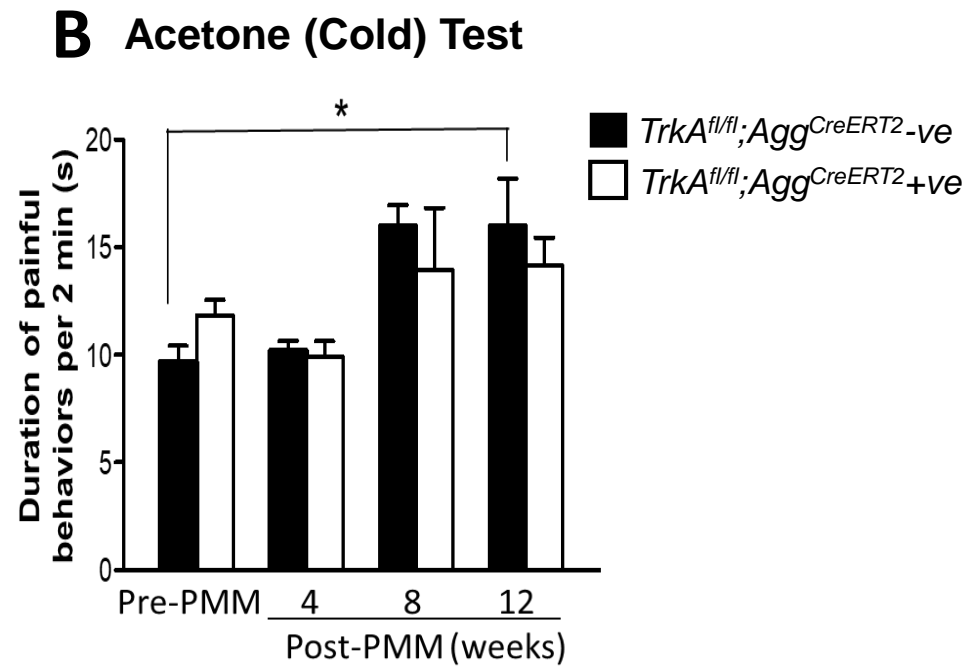

Supplement: Supplementary file 1 [file ijms-23-12076-s001.zip › ijms-1939378-supplementary.pdf]
